# Why small males have big sperm: dimorphic squid sperm linked to alternative mating behaviours

**DOI:** 10.1186/1471-2148-11-236

**Published:** 2011-08-10

**Authors:** Yoko Iwata, Paul Shaw, Eiji Fujiwara, Kogiku Shiba, Yasutaka Kakiuchi, Noritaka Hirohashi

**Affiliations:** 1 Institute of Biological, Environmental and Rural Sciences (IBERS), Aberystwyth University, Penglais, Aberystwyth, SY23 3DA, UK; 2 Atmosphere and Ocean Research Institute, University of Tokyo, Kashiwa, Chiba 277-8564, Japan; 3 Documentary Channel Co. Ltd., Kawaguchi, Saitama 333-0844, Japan; 4 Shimoda Marine Research Center, University of Tsukuba, Shimoda, Shizuoka 415-0025, Japan; 5 Science and Education Center, Ochanomizu University, Bunkyo, Tokyo 112-8610, Japan; 6 School of Humanities and Sciences, Ochanomizu University, Bunkyo, Tokyo 112-8610, Japan

## Abstract

**Background:**

Sperm cells are the target of strong sexual selection that may drive changes in sperm structure and function to maximize fertilisation success. Sperm evolution is regarded to be one of the major consequences of sperm competition in polyandrous species, however it can also be driven by adaptation to the environmental conditions at the site of fertilization. Strong stabilizing selection limits intra-specific variation, and therefore polymorphism, among fertile sperm (eusperm). Here we analyzed reproductive morphology differences among males employing characteristic alternative mating behaviours, and so potentially different conditions of sperm competition and fertilization environment, in the squid *Loligo bleekeri*.

**Results:**

Large consort males transfer smaller (average total length = 73 μm) sperm to a female's internal sperm storage location, inside the oviduct; whereas small sneaker males transfer larger (99 μm) sperm to an external location around the seminal receptacle near the mouth. No significant difference in swimming speed was observed between consort and sneaker sperm. Furthermore, sperm precedence in the seminal receptacle was not biased toward longer sperm, suggesting no evidence for large sperm being favoured in competition for space in the sperm storage organ among sneaker males.

**Conclusions:**

Here we report the first case, in the squid *Loligo bleekeri*, where distinctly dimorphic eusperm are produced by different sized males that employ alternative mating behaviours. Our results found no evidence that the distinct sperm dimorphism was driven by between- and within-tactic sperm competition. We propose that presence of alternative fertilization environments with distinct characteristics (i.e. internal or external), whether or not in combination with the effects of sperm competition, can drive the disruptive evolution of sperm size.

## Background

Postcopulatory sexual selection can occur in situations where females mate with more than one male and ejaculated spermatozoa compete for fertilization [1]. Because sperm traits have a direct impact on fertilization success, they are subject to strong postcopulatory sexual selection forces in polyandrous species [2]. Theoretical models suggest that the pressures of sperm competition and cryptic female choice will drive sperm evolution towards an optimal morphology [3]. In support of this prediction inter-male variation of sperm morphology in birds is negatively associated with the level of sperm competition [4]. Similarly, in *Drosophila *sperm length coevolves with the length of the female reproductive tract as paternity bias is selected by female morphology [5], and so an optimal sperm morphology that fits with the majority of female reproductive morphologies would be selected, resulting in reduced intra-specific diversity in sperm morphology.

Sperm competition theory predicts that sperm size is influenced by the intensity of sperm competition among males [reviewed in [6]], with either larger or smaller sperm favoured depending on the underlying assumptions [3]. Empirical studies support this prediction in a range of taxa including insects [7], amphibians [8], fish [9,10], birds [11] and mammals [12,13], although recent studies have found no clear link between sperm size and sperm competition intensity, when considered the phylogenetic relationships [14-16]. However, definitive tests of the predictions from sperm competition theory using empirical data have been mostly restricted to comparisons between related species with different levels of polyandry or gonadosomatic index. Intra-specific tests are possible where alternative male reproductive tactics, in which consort males guard females and sneaker males steal fertilizations from consort males, result in biased sperm competition risk among males [17]. Previous studies have tested if differing sperm competition risk leads to different sperm size between tactics, but only one study supported the prediction that sneaker males have longer sperm than consort males [18]. However, even in this study the difference in sperm sizes is thought to be attributable to more variable sperm length within consort males and due to a few consort individuals having unusually short sperm [19].

Aside from the effects of sperm competition, adaptation to fertilization environments can be predicted to have an impact on the evolution of sperm traits. Species displaying internal or external fertilization will employ quite different mating strategies, and therefore sperm traits. Differing physiological conditions in fertilization site in terrestrial or aquatic mating habitats have been shown to influence the evolution of sperm size in frogs [8]. As sperm evolution theory has mainly been driven by comparative analysis among related species, the effects of differences in fertilization environments in driving sperm trait evolution have been often overlooked in previous studies.

**Figure 1:**
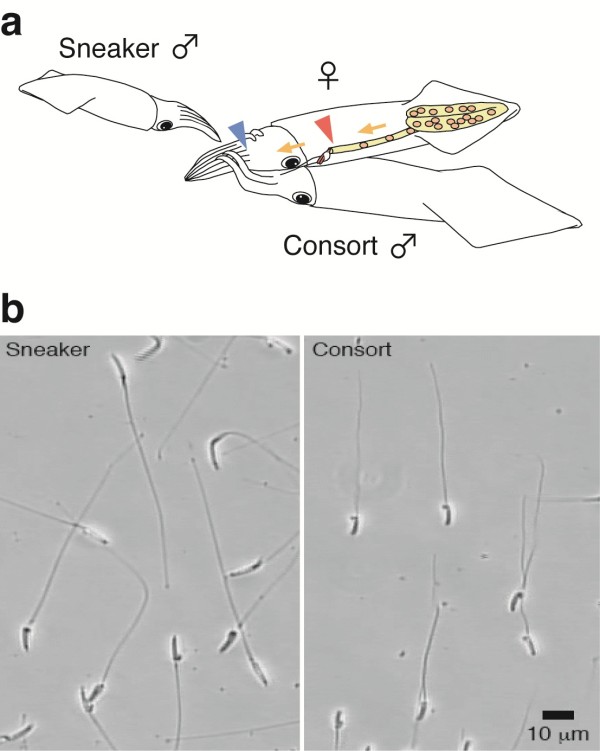
**Two types of male mating behaviour and sperm size**. (a) Alternative reproductive tactics by males and sperm storage sites in the female body. The externally located seminal receptacle (blue) and the internal oviduct opening (red) are the targets for sperm transfer by smaller sneaker and larger consort males, respectively. Yellow arrows indicate the route for egg passage. (b) Sperm dimorphism in the squid *Loligo bleekeri*. A representative DIC image of spermatozoa collected from consort (right) and sneaker (left) males (scale bar, 10 μm).

The squid *Loligo bleekeri *is an ideal species to examine the evolution of sperm morphology under sexual selection within a species, because it exhibits alternative reproductive tactics that create discretely different fertilization conditions among males within a single spawning episode [20]. Females store spermatozoa in two separate locations in/on their bodies, whereas individual males transfer spermatozoa to one or other of these sites according to a mating tactic related to their body size, similar to that observed in other *Loligo *species [21,22]; Figure 1a]. Larger "consort" males compete with other males and court females using body colouring displays. Successful consorts mate with females in a parallel position, place spermatophores inside the female's oviduct opening, and guard the female until she spawns egg strings. Smaller "sneaker" males show few male-competition and courtship behaviours, but instead rush into an established consort male and female pair, mate in a head-to-head position and place their spermatophores on to the female's external body surface near the sperm storage organ (the seminal receptacle) located below the mouth. Both consort and sneaker males can achieve fertilization during a spawning event, although fertilization success is higher for consort males [23]. Adult males show discrete spermatophore dimorphism, where larger males produce significantly longer spermatophores than smaller males [24], spermatophore size variation is small within individuals, and no individual has both types of spermatophore simultaneously. Spermatophore dimorphism is linked also to female sperm storage sites (large within the oviduct, small around the seminal receptacle), confirming a tight association with alternative male mating tactics. The life span of this species is one year, with a single and short terminal reproductive period [25]. Although it is not known to what extent either genetic or environmental factors determine male morphology or mating tactic, individuals are thought to be specialized for one mating tactic and ontogenetic transition from small sneaker to large consort is unlikely.

Due to the process of egg laying in *L. bleekeri*, insemination and fertilization can therefore occur in two sites under different environmental conditions: a string of eggs is extruded from the oviduct (inside the mantle cavity) where it is exposed to consort sperm inside the oviduct (i.e. internal fertilization conditions), and the female pulls the egg string through her siphon and into position within her arm crown around the mouth, where it is exposed to sneaker sperm (i.e. external fertilization conditions), before she deposits the egg string onto the sea bed. The different fertilization environments can be predicted to lead to different optimal outcomes for sperm size evolution. Here we investigate the first example of dimorphic fertile sperm within a species, which is associated with alternative male mating tactics and alternative fertilisation environments.

## Results

### Sperm size

**Figure 2:**
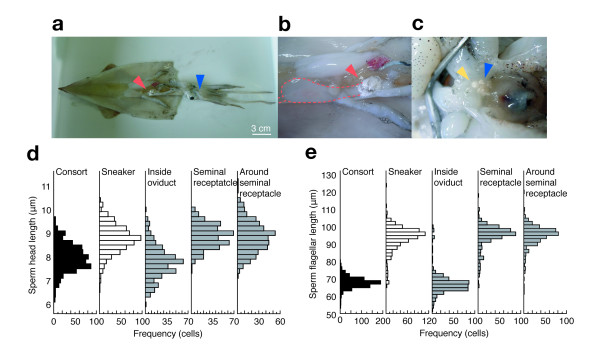
**Size distribution of sperm recovered from males and females**. (a) Anatomical view of sperm storage sites on the female where spermatophores are attached by consort (red arrowhead) and sneaker (blue arrowhead) males. (b) The spermatophores are attached by consort males to the inside wall of the oviduct (red broken line) at the posterior end. The oviduct was dissected to show its inside where sperm mass were attached. (c) Sneaker males attach spermatophores to the skin surface (blue arrowhead) adjacent to the seminal receptacle (yellow arrowhead) located under the mouth. Sneaker sperm released from sperm masses are transported to the seminal receptacle by an unknown mechanism. Histograms of head length (d) and flagellar length (e) of sperm collected from sperm masses in the oviduct (Inside oviduct), inside the seminal receptacle and from around the seminal receptacle periphery (Around seminal receptacle), compared with sperm recovered from consort and sneaker spermatophores.

To assess differences in sperm morphology among males, we collected and measured the size of spermatozoa from males defined as consorts or sneakers. We found that sneaker males produce sperm with a longer head and flagellum than those of consort males (sneaker: head length = 8.47 ± 0.60 μm, flagellum length = 90.5 ± 7.20 μm, n = 600. consort: head length = 7.67 ± 0.59 μm, flagellum length = 64.9 ± 3.22 μm, n = 600. Linear Mixed Models (LMMs): head χ2 = 55.41, P < 0.01; flagellum χ2 = 93.38, P < 0.01. Figures 1b, 2). The size distribution of sperm stored in the seminal receptacle (head length = 8.56 ± 0.50 μm, flagellum length = 91.7 ± 6.95 μm, n = 400) and in sperm masses attached around the female's mouth (head length = 8.54 ± 0.67 μm, flagellum length = 91.8 ± 6.49 μm, n = 400) were statistically indistinguishable from sperm in sneaker spermatophores (head length = 8.47 ± 0.60 μm, flagellum length = 90.5 ± 7.20 μm, n = 600; LMMs, head: χ^2 ^= 1.68, *P *= 0.43; LMMs flagellum: χ^2 ^= 1.35, *P *= 0.51; Figure 2). The size distribution of sperm stored in oviduct (head length = 7.50 ± 0.68 μm, flagellum length = 64.3 ± 6.55 μm, n = 400) was statistically indistinguishable from sperm in consort spermatophores (head length = 7.67 ± 0.59 μm, flagellum length = 64.9 ± 3.22 μm, n = 600; LMMs, head: χ^2 ^= 1.92, *P *= 0.17; LMMs flagellum: χ^2 ^= 0.30, *P *= 0.58; Figure 2), suggesting that the sperm dimorphism is closely related with the alternative sperm storage sites.

### Fertility

**Figure 3:**
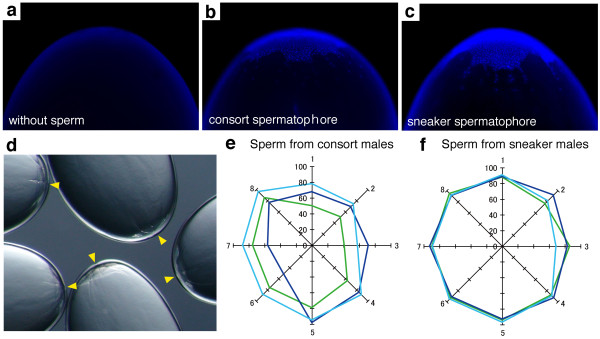
**Fertilization success by consort and sneaker sperm**. (a-c) Mature oocytes were inseminated without sperm as a negative control (a), with sperm from consort spermatophores (b) and from sneaker spermatophores (c), and then cultured 48 hours and stained with DAPI. (d) A DIC image of developing embryos after 24 hours of insemination: arrowheads point to the animal pole where cleavage planes appear along with the animal-vegetal axis. (e, f) Fertilization rates in oocytes from eight females (1-8) inseminated with sperm from consort (e) or sneaker (f) spermatophores. Each colour-coded line represents one male.

To examine if both types of sperm are competent for fertilization, we carried out an *in vitro *fertilization assay using ovulated fresh oocytes retrieved from the oviduct (Figure 3). Control oocytes that were not inseminated (Figure 3a) showed no sign of embryo development after 24 hours, whereas oocytes inseminated with sperm collected from consort spermatophores (Figure 3b), sneaker spermatophores (Figure 3c), and from sperm masses (spermatangia) retrieved from the female's oviduct or seminal receptacle (data not shown) showed multinuclear staining as a result of discoidal cleavage at the animal pole (Figure 3d). Success rates of *in vitro *fertilization reached 41-98% in combinations between the same 8 females and either 3 consort or 3 sneaker males (Figure 3e, f), suggesting that sperm from both male types were capable of fertilization and genetically compatible with the females.

### Motility and number of sperm

We measured swimming velocity of both type of sperm and found no difference between sneakers and consorts (consort curvilinear velocity = 167.7 ± 36.9 μm/s, n = 6, 1421 cells; sneaker velocity = 167.1 ± 33.5 μm/s, n = 3, 619 cells; Mean ± SE, Student's t-test, *P *= 0.745).

**Figure 4:**
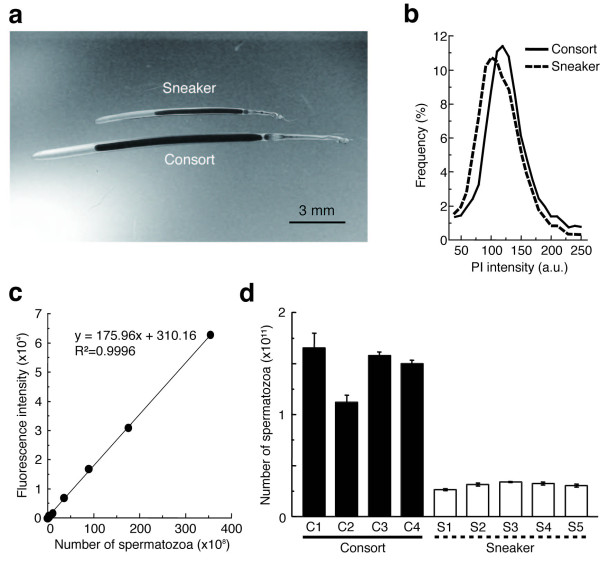
**Determination of sperm number within a single spermatophore**. (a) Spermatophores from consort and sneaker males. (b) The relative amount of sperm DNA defined as the propidium iodide fluorescence intensity by flow cytometric analyses from consort (n = 10) and sneaker (n = 9) males. (c) A standard calibration curve was generated by plotting the fluorescence intensity versus sperm concentrations. (d) Estimated sperm number per spermatophore of consort (n = 4) and sneaker (n = 5) individuals. Each bar represents the mean ± s.e.m. of five spermatophores from an individual after the weighted calibration from (b) and (c).

The number of spermatozoa within a single spermatophore was different between consorts and sneakers: 1.46 ± 0.12 × 10^11 ^cells in consorts and 3.09 ± 0.13 × 10^10 ^cells in sneakers (consort, n = 4; sneaker, n = 5; Mean ± SE, Figure 4).

## Discussion

This is the first reported case of discrete dimorphism of fertile sperm (eusperm) exhibited between individuals within a species. A few previous studies have suggested that sperm morphology is influenced by different sperm competition risks associated with different male mating tactics [18,26], although the observed differences in sperm morphology between tactics are small compared to large variation within a tactic. Why is much greater (and highly significant) divergent evolution of sperm morphology evident among males of the squid *L. bleekeri*? First, it is possible that sperm competition does not operate under a "fair raffle" system [27] between consort and sneaker males due to storage site-dependent sperm precedence towards consorts (between-tactic sperm competition). As consort sperm, released in the oviduct, have access to the oocytes earlier than sneaker sperm, stored in the external location, sneaker sperm might be predicted to possess increased swimming speed (and so size) to offset the consort sperm advantage. Increased motility can be an effective strategy as time from insemination until sperm-egg fusion may be sufficiently long to allow sneaker sperm to compete, as observed in the frog *Rana temporaria *in which sneaker (pirate) males ejaculate onto a deposited egg mass after it has already been inseminated by a guarding male, but still achieve a mean fertilization success of 24.1% [28]. A positive relationship between sperm size and swimming velocity has been found in many animals [9,29], although there are a few exceptions [10,14], and sperm swimming velocity is positively related to fertilization success in species both with internal and external fertilization [30,31]. We tested *L. bleekeri *sperm swimming velocity and found no difference between sneakers and consorts, suggesting that this factor is unlikely to explain the size dimorphism.

A second possible explanation for dimorphic sperm would be strong selection among sperm from sneaker males competing for space in the seminal receptacle (within-tactic sperm competition). Larger sperm would have an advantage in occupying spaces within the seminal receptacle to exclude, and so outcompete, smaller sperm [5,32]. In this case, the size distribution of sperm stored in the seminal receptacle would be expected to be biased upwards compared to that found in the original sperm populations. Contrary to this prediction, the size distribution of sperm stored in the seminal receptacle was not different from that in sneaker spermatophores or in sperm masses attached around the female's mouth, suggesting that this factor also is unlikely to explain the size dimorphism

A third explanation for dimorphic sperm would be divergent selection pressures on sperm from different male mating types as a consequence of adaptation to different fertilization environments (internal versus external). Males adopting different mating tactics are expected to maximize fitness in different ways, in the context of reproductive energy expenditure as a trade-off between sperm size and number. In some examples of consort versus sneaker male strategies, sneaker males produce relatively larger numbers of sperm than consorts to offset the positional mating advantage of consorts [17,18]. Contrary to this expectation, in *L. bleekeri *the number of spermatozoa within a single spermatophore was estimated to be ~5-fold greater in consorts than in sneakers (Figure 4). The different sperm size versus number strategies employed by sneakers and consorts, running contrary to expectations under simple sperm competition, suggests that fertilization environment (the other major difference between sneaker and consort strategies) may be an important factor in determining the sperm size/number trade-off in this species. Aside from the obvious factor of water movement (i.e. risk of sperm dilution), there may be many differences between external and internal fertilization environments (such as salinity, viscosity, pH and concentrations of gases and nutrients) that may affect fertilization success by different sized sperm. Although there have been a number of cases reported, across diverse taxa, of species exhibiting alternative male mating tactics, there has been no clear evidence of sperm dimorphism between sneaker and consort males [18,33,34]. The common factor among these previous studies is that despite sperm from each tactic facing different sperm competition conditions arising from different male competition behaviours, courtship behaviours, mating order, mating duration and sperm expenditure [33], how and where released sperm meet with eggs (i.e. fertilization environments) are basically the same among competing males. Given the accepted importance of sperm competition in the evolution of male mating strategies (including individual and sperm morphology, physiology and behaviour), it is likely that sperm competition is also a strong selective agent in the evolution of sperm and ejaculate characteristics in *L. bleekeri*, perhaps in optimizing these characteristics for each insemination/fertilization site. However, viewing previous observations together with the data presented here, it can be proposed that fertilization environment has a predominant adaptive significance for sperm size diversification in *L. bleekeri *and other species.

## Conclusions

In conclusion, postcopulatory sexual selection under strong constraints associated with alternative mating tactics can drive discrete sperm polymorphism. Although alternative mating tactics can create differences in sperm competition risk, in the squid *L. bleekeri *they also produce different fertilization opportunities conditioned by the internal versus external environment. At the moment the mechanisms by which sperm polymorphism has evolved remain elusive. However, our study illustrates that *L. bleekeri *constitutes a fascinating and suitable model system for answering such questions in evolutionary biology, behavioural ecology and sexual reproduction.

## Methods

### Handling of animals

For sperm size measurements and bioassays, mature *Loligo bleekeri *were collected at Miura (Sagami-bay, Kanagawa) or Matsumae (southeast Hokkaido Island), Japan, and transported to the laboratory at 4°C within 48 h. Adult males produce spermatophores, which are cylindrical capsules containing mature sperm. Consort males and sneaker males can be distinguished by measuring the mantle and spermatophore lengths. Spermatophore length shows discrete dimorphism associated with mating tactics [[24]; Figure 4a]. To allay the suggestion that the two types of males may represent a cryptic species complex, we sequenced a 776 base pair region of the mitochondrial cytochrome c oxidase subunit I (COI) gene (Additional file 1). Haplotype frequencies and genetic distances among 27 consort and 29 sneaker males, sampled from both study populations at Miura and Matsumae, show no significant genetic differences, suggesting no reproductive isolation between consort and sneaker male populations.

For artificial fertilization experiments, we used live mature squid commercially fished at Miura, Japan, in April and May. Each female was put in a plastic bag filled with fresh seawater, saturated with O_2 _and transported to the laboratory within 30 min. Squid were maintained at 15°C in aquaria at the Misaki Marine Biological Station, University of Tokyo.

### Sperm length measurements

Spermatozoa were released from spermatophores in a 1.5 ml tube containing 200 μl of seawater, followed by 1-h incubation on ice to recover the concentrated sperm suspension. A volume of 100 μl of the upper layer, which contained enriched swim-up sperm and less cell debris, was transferred to a fresh 1.5 ml tube and fixed with an equal volume of 4% formaldehyde-containing seawater. The samples were observed by DIC microscopy (Nikon) and photomicrographs were taken at 200× magnification using a CCD camera (Keyence). DIC images were analyzed with NIH ImageJ to measure head and flagellar length. We measured 20 sperm per individual from 30 consort and 30 sneaker males. During the breeding season, females often carry the sperm masses attached by males to specific sites on their body. The sperm masses found in different parts of the female body (inside the oviduct, and inside and outside of the seminal receptacle below the mouth) were isolated surgically, minced in a 1.5 ml test tube containing 200 μl seawater and subjected to the same procedure described above. Twenty samples were collected from each sperm storage site, and 20 sperm were measured per sample.

### Sperm fertility test by in vitro artificial insemination

We tested fertilization competence in combinations between 3 females and 8 consort and 8 sneaker males collected at Miura. The following method was developed by modifying a protocol previously reported for other squid species [35]. Spermatophores were removed from the male's Needham's sac (male reproductive accessory organ, where mature spermatophores are stored) and were stimulated to ejaculate the sperm mass. Spermatozoa released naturally from the sperm mass were motile, and were used for in vitro fertilization assays. Mature oocytes were obtained from the oviduct and placed in a 35-mm diameter Petri dish in the absence of seawater. Approximately 200 oocytes per dish were inseminated with 100~200 μl of sperm suspension followed by gentle stirring with a plastic spatula. Five minutes after insemination, the dishes were filled with seawater and kept at 15°C. After 30 min, excess sperm were removed and replaced with fresh seawater several times as a washing step, and then incubated at 15°C. Fertilization success was determined by the presence or absence of cleavage planes in the animal hemisphere of the egg after 12-h incubation. At least 50 eggs were scored using a stereomicroscope. Normal embryonic development was confirmed by Hoechst 33342 stain (5 μg/ml) of 4% formaldeyde-fixed specimens after 24-h culture and photographed under a UV fluorescent microscope.

### Sperm motility

Sperm swimming velocity was measured by SMAS (Sperm Motility Analysis System, Ditect, Tokyo, Japan), which automatically tracks mobile sperm under the microscope and calculates motility parameters (curvilinear velocity along sperm swimming path per sec).

### Sperm number in a spermatophore

We measured sperm number contained within the spermatophores using fluorescence intensity of stained sperm lysates. To examine the integrity and heterogeneity of nuclear DNA in the sperm population, microscopic and flow-cytometric analyses were performed. Spermatozoa released from spermatophores were fixed with an equal volume of 4% formaldehyde-containing seawater (pH 7.9), rinsed twice with seawater after 30-min incubation, and stained with propidium iodide (PI) in seawater at a final concentration of 50 μg/ml for 30 min. Fluorescence microscopy revealed that PI-staining was specific to the sperm head and homogenous within the population. PI-stained spermatozoa were then suspended in 300 μl of PBS and flow-cytometric analysis was performed by acquiring at least 15,000 gated events per sample obtained from consort (n = 10) and sneaker (n = 9) males (Ex. 488 nm, Em. 607 nm; Cell Lab Quanta SC, Beckman Coulter, Tokyo, Japan).

The sperm masses discharged from single spermatophores, by cutting the center of the amber ejaculatory apparatus, were recovered in 1.5-ml tubes. Thereafter, they were dissolved in 1 ml lysis buffer (10 mM Tris-HCl pH 9.5, 0.1 mg/ml proteinase K) at 37°C with vigorous shaking for 12 h. The lysates were then diluted (2- to 10-fold) with lysis buffer to obtain a series of different sperm concentrations and a standard calibration curve between the fluorescence intensity versus sperm concentrations. Analysis of DNA content in 100 μl aliquots of the sperm lysate was performed with a genomic DNA quantitation kit (Molecular Probes, FluoReporter Blue Fluorometric dsDNA Quantitation kit) according to the manufacturer's protocol. A standard curve was made using a series of lysate dilutions and calibrated against the original sperm suspensions using a hemocytometer.

### Statistical analyses

Linear mixed models (LMMs) are used when analyzing hierarchical data assuming normally distributed errors, for example to account for repeat sampling of the same individuals [36]. We constructed LMMs using sperm size as dependent variable and category of sperm populations (consort vs. sneaker) as fixed effect. We also constructed separate LMMs using categories (consort vs. sperm stored in oviduct, and sneaker vs. sperm stored in seminal receptacle vs. sperm in masses attached around seminal receptacle) as fixed effect, to confirm if the sperm in each storage site on the female is associated with the alternative male mating tactics. Sample identity (male individual or sperm mass attached to female) was set as a random effect in the models. The significance of the fixed effects on dependent variables, such as mating tactic or sperm storage site, was assessed with the likelihood ratio test, using the log-likelihood of the test model (including fixed effect) and the null model (without fixed effect). We used the ''lme4'' package in R^® ^2.7.1 software [37] to run the LMMs analyses.

## Authors' contributions

YI and NH designed research; YI, EF, KK, YK and NH performed research; YI, PS, KS, YK and NH analyzed data; and YI, PS and NH wrote the paper. All authors read and approved the final manuscript.

## Supplementary Material

Additional file 1 **Mitochondrial DNA analysis confirming that two types of males are not cryptic species or subpopulations**. Click here for file
