# Supplementary material for: Why small males have big sperm: dimorphic squid sperm linked to alternative mating behaviours
Source: BMC Evol Biol. 2011 Aug 10;11:236. doi: 10.1186/1471-2148-11-236 (PMC3176235; doi:10.1186/1471-2148-11-236)
Supplement: Additional file 1 — Mitochondrial DNA analysis confirming that two types of males are not cryptic species or subpopulations. [file 1471-2148-11-236-S1.DOC]

**Additional file 1 – Mitochondrial DNA analysis confirming that two types of males are not cryptic species or subpopulations**

**Additional materials and methods**

Mitochondrial DNA (mtDNA) was extracted from muscle tissue of consort and sneaker males using a genome DNA isolation kit (TaKaRa, FastPure DNA kit). A 776-bp coding region in the cytochrome oxidase subunit I (COI) gene was PCR amplified with primers ATGCGATGACTATTTTCCACA (sense) and GAGTGATGAGATACAATATGTG (antisense), then sequenced. A total of 56 (27 consort and 29 sneaker) males, sampled from both study populations at Miura and Matsumae, were examined for mtDNA COI sequence variation, revealing 12 haplotypes as shown in Table S1. Two common haplotypes (LBCOI-1 and LBCOI-4) are equally distributed in both consort and sneaker populations and there are no significant genetic differences between the two groups in terms of haplotype frequencies (exact test *P* = 0.22265; *F_ST_*, = -0.012, *P* = 0.173; Arlequin v. 3.11) [S1] or within- versus between-group genetic divergence (both = 0.00374; DAMBE) [S2]. These results suggest that reproductive isolation is not present between consort and sneaker males, i.e. they do not represent cryptic species or subpopulations.

**Additional results**

Table S1. Single nucleotide polymorphism (SNP) in a 559-bp coding region (116-774 bp from the start codon) of the mitochondrial *CO-I* gene.


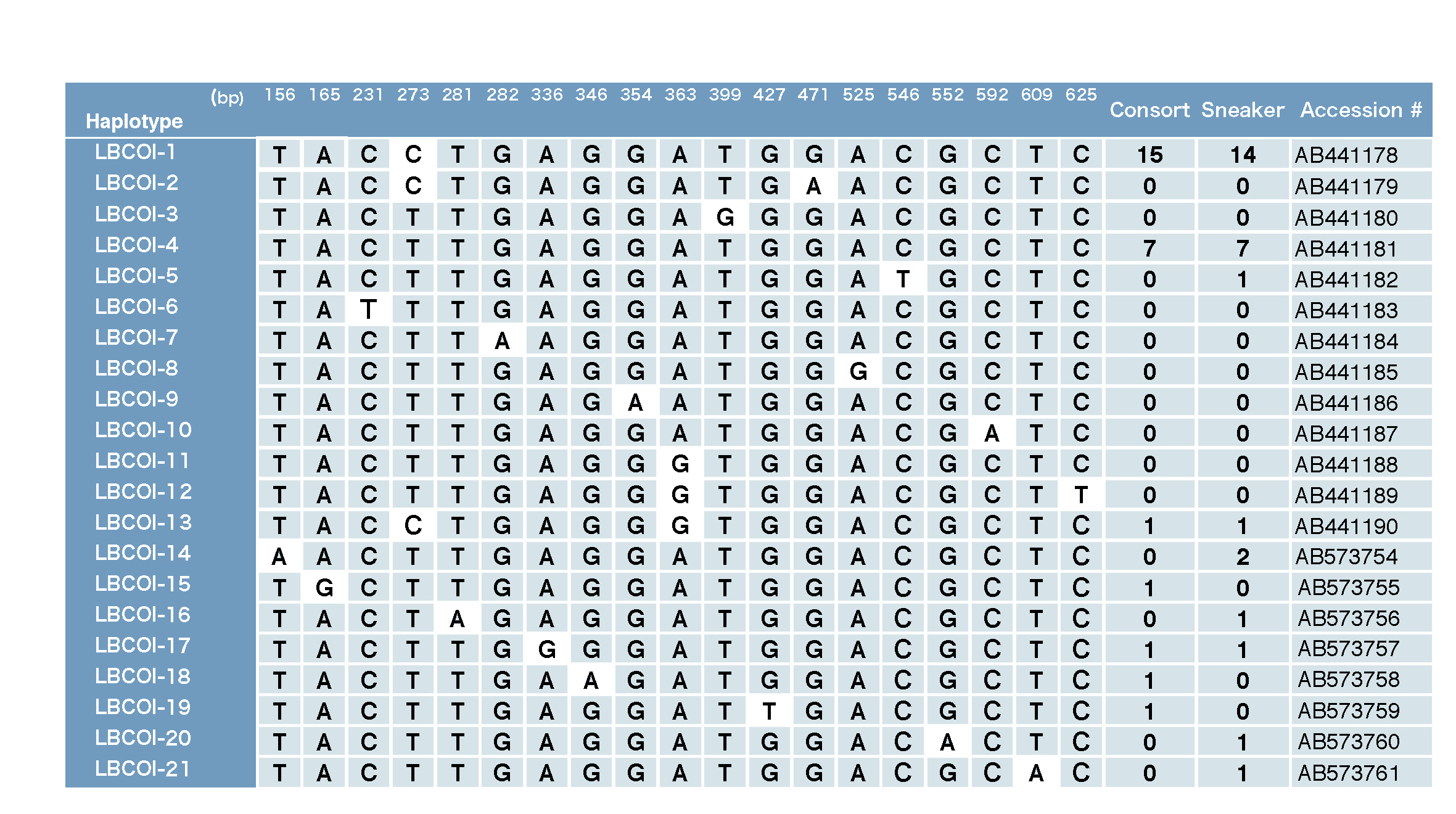


Sequences of 13 previously reported haplotypes (LBCOI-1 to LBCOI-13) and 8 new haplotypes identified in this study (LBCOI-14 to LBCOI-21) are presented for comparison. Frequencies of the haplotypes were analyzed with 27 consort and 29 sneaker male individuals in this study. Consort and sneaker populations show no difference in genetic composition: genetic distance (S1) is identical for within- and between-group comparisons (both = 0.00374; DAMBE, S2).

**Additional references**

S1 Excoffier L, Laval G, Schneider S: **Arlequin ver. 3.0: An integrated software package for population genetics data analysis.** *Evolutionary Bioinformatics Online* 2005, **1**: 47-50.

S2 Xia X, Lemey P: **Data exploration and tetrapod phylogeny using DAMBE.** In *The Phylogenetic Handbook: A Practical Approach to DNA and Protein Phylogeny.* 2nd edn. Ed. Lemey P, Salemi M, Vandamme A-M. Cambridge: Cambridge University Press; 2009: 611-629.
